# A Molecular Signal Integration Network Underpinning *Arabidopsis* Seed Germination

**DOI:** 10.1016/j.cub.2020.07.012

**Published:** 2020-10-05

**Authors:** Hao Xu, Ourania Lantzouni, Tonko Bruggink, Rene Benjamins, Frank Lanfermeijer, Katherine Denby, Claus Schwechheimer, George W. Bassel

**Affiliations:** 1 School of Life Sciences, University of Warwick, Coventry CV4 7AL, UK; 2 Plant Systems Biology, Technical University of Munich, 85354 Freising, Germany; 3 Department of Biology, University of York, York YO10 5DD, UK; 4 Applied Physiology, Syngenta Seeds B.V., P.O. Box 2, NL-1600AA Enkhuizen, the Netherlands

**Keywords:** seed germination, cell expansion, network, gibberellic acid, DELLA, signal integration, embryo, nitric oxide

## Abstract

Seed dormancy is an adaptive trait defining where and when plants are established. Diverse signals from the environment are used to decide when to initiate seed germination, a process driven by the expansion of cells within the embryo. How these signals are integrated and transduced into the biomechanical changes that drive embryo growth remains poorly understood. Using *Arabidopsis* seeds, we demonstrate that cell-wall-loosening *EXPANSIN* (*EXPA*) genes promote gibberellic acid (GA)-mediated germination, identifying *EXPA*s as downstream molecular targets of this developmental phase transition. Molecular interaction screening identified transcription factors (TFs) that bind to both *EXPA* promoter fragments and DELLA GA-response regulators. A subset of these TFs is targeted each by nitric oxide (NO) and the phytochrome-interacting TF PIL5. This molecular interaction network therefore directly links the perception of an external environmental signal (light) and internal hormonal signals (GA and NO) with downstream germination-driving *EXPA* gene expression. Experimental validation of this network established that many of these TFs mediate GA-regulated germination, including *TCP14/15*, *RAP2.2/2.3/2.12*, and *ZML1*. The reduced germination phenotype of the *tcp14 tcp15* mutant seed was partially rescued through ectopic expression of their direct target *EXPA9*. The GA-mediated control of germination by *TCP14/15* is regulated through *EXPA*-mediated control of cell wall loosening, providing a mechanistic explanation for this phenotype and a previously undescribed role for TCPs in the control of cell expansion. This network reveals the paths of signal integration that culminate in seed germination and provides a resource to uncover links between the genetic and biomechanical bases of plant growth.

## Highlights

• The network linking integration of environmental signals to seed growth is mapped
• *EXPANSIN* gene expression is redundantly regulated and promotes GA-mediated germination
• The TCP14 transcription factor directly regulates *EXPANSIN9* expression
• The *tcp14/15* germination phenotype is complemented by *EXPANSIN9* expression

## Teaser

Seeds use a range of environmental cues to decide when to commence germination. Xu et al. report the molecular interaction network used to integrate inputs into growth-promoting *EXPANSIN* gene expression. Functional validation defines *EXPANSIN*s as downstream factors driving germination and TCP14/15 as transcription factors mediating their expression.

## Introduction

The development of plants is highly plastic, being capable of change in response to their environment [1]. Rather than making use of individual signals from the environment, complex combinations of inputs are perceived and integrated in order to make developmental decisions. An example of complex signal integration is observed in seed dormancy. This adaptive trait enables plants to move [2] by imposing a growth arrest upon the enclosed embryo [3]. The decision to break dormancy and restart growth is influenced by a variety of signals from the environment including temperature, light quality, smoke, and others [4]. These multiple signals must be perceived, integrated, and turned into a single decision to restart embryo growth. How these diverse environmental inputs are integrated and transduced to stimulate embryo growth remains unknown.

The seed-to-seedling transition in *Arabidopsis* is principally driven by cell expansion [5, 6]. Immediately preceding this induction of cell growth is the expression of diverse gene families that encode proteins that modify the cell wall [7, 8, 9]. These include expansins (*EXPA*s) [10], xyloglucan endo-transglycosylases (XETs) [11], pectin methylesterases (PMEs) [12], and polygalacturonases (PGs) [13]. The functional contribution of each individual class of cell-wall-modifying protein toward the promotion of germination remains unclear.

Both germination and cell-expansion-associated gene expression are promoted by the hormone gibberellic acid (GA). The action of this hormone has been demonstrated to occur in multiple sites within seeds, promoting cell expansion in both the embryo [6] and the endosperm [14] in *Arabidopsis*. A necessity for GA in the seed-to-seedling transition is not present because embryos from GA-deficient genotypes grow into stunted seedlings, whereas intact seeds in this species depend on the hormone for germination to occur [15]. Evidence of bi-directional communication between the embryo and endosperm is proposed to coordinate growth across these tissues [16, 17].

GA responses are repressed by DELLA proteins [8, 18]. DELLAs have been proposed to act by physically interacting and inhibiting the activity of transcription factors (TFs) [19]. DELLAs also integrate different signals from the environment, including light and temperature, to control plant growth and development [20]. The mechanistic basis as to how signals from the environment are transduced via GA and DELLAs into the gene expression driving cell expansion and germination remains unknown.

Interaction mapping provides a powerful means to uncover relationships between molecular entities as well as system-level properties, and to identify previously uncharacterized regulators of developmental processes. Systematic mapping of protein-protein interactions in plants has identified regulatory hubs as targets of pathogen effectors [21], established protein-DNA interactions as identified novel regulators of vascular development [22], uncovered the architecture of nitrogen assimilation [23], and described the cistrome-binding landscape of TFs in *Arabidopsis* [24].

This study makes use of interaction mapping to uncover the molecular network used by seeds to integrate multiple signals from the environment and transduce this into growth-promoting gene expression and germination. This establishes direct molecular links between the perception of environmental signals and the downstream gene expression driving a developmental phase transition in plants.

## Results

### *EXPA* Expression Promotes GA-Mediated Seed Germination

A range of gene families associated with cell-wall modification is induced during *Arabidopsis* seed germination [7, 25, 26]. In light of a clearly demonstrated role in promoting plant cell expansion [10] and the implication that they participate in the germination process [14, 26, 27], we investigated the role of *EXPA* genes in GA-mediated embryo growth.

**Figure 1:**
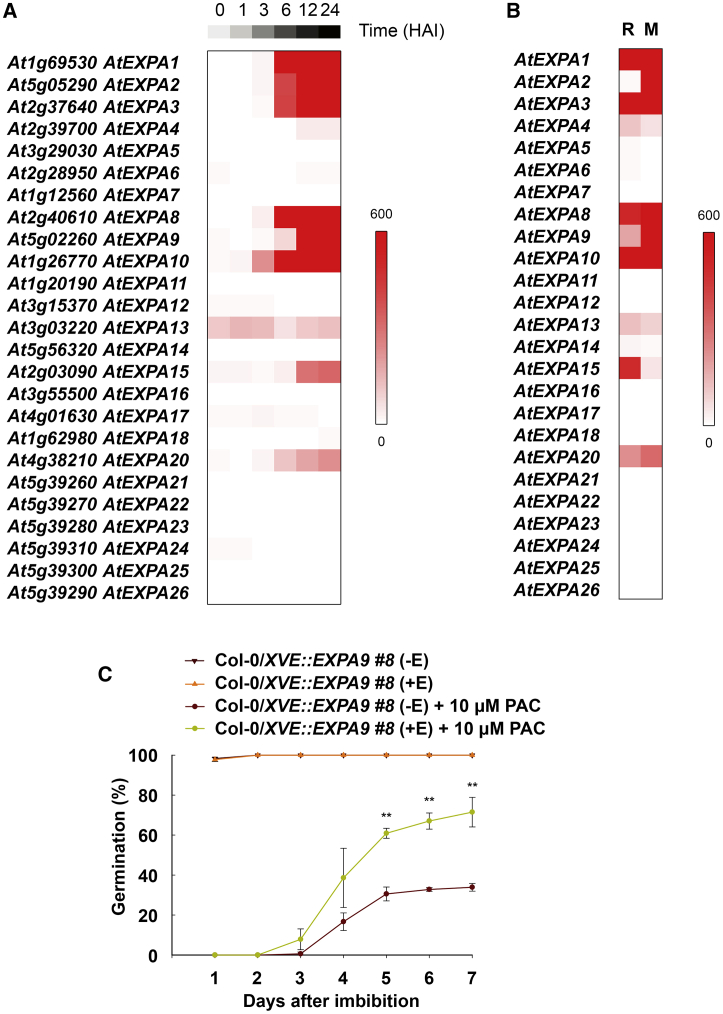
*EXPA* Expression and Function during Seed Germination (A) Heatmap showing expression of the *EXPA* gene family in germinating seeds at different time points after imbibition (0, 1, 3, 6, 12, and 24 h) [7]. (B) Expression of the *EXPA* gene family in the radicle (R) and micropylar endosperm (M) at 16 h after imbibition [25]. (C) Germination of *XVE::EXPA9* seeds on water and in the presence of 10 μM GA-synthesis inhibitor PAC. Ectopic expression of *EXPA9* is induced after the application of 30 μM β-estradiol (+E). Data are expressed as mean value ± standard deviation (SD) (n ≥ 50 seeds per biological replicate). See also Figure S1.

In *Arabidopsis*, the α-*EXPA* gene family consists of 26 members [28]. Publicly available gene expression data indicate 8 of these are induced during seed germination (Figure 1A) [7]. Of these 8 induced genes, only *EXPA2* is specific to the endosperm and not present in the embryo (Figure 1B) [14, 25, 26]. The subset of 7 genes including *EXPA1*, *EXPA3*, *EXPA8*, *EXPA9*, *EXPA10*, *EXPA15*, and *EXPA20* represents embryo-induced *EXPA* family members.

The induction of *EXPA* genes during seed germination in response to GA has been reported previously [8, 26, 29]. The functional role this *EXPA* gene expression plays in the control of germination, however, remains poorly defined. We examined this by creating an inducible construct consisting of *XVE::EXPA9*, enabling high-level expression in response to estrogen application [30] (Figure S1B).

Seed germination is reduced in the presence of the GA-synthesis inhibitor paclobutrazol (PAC) (Figure 1C) [31]. Ectopic induction of *EXPA9*, however, partially restored this GA-limited germination phenotype (Figures 1C and S1A). This demonstrates *EXPA* expression supports embryo growth under GA-limiting conditions, and presents this gene family as downstream molecular targets promoting the seed-to-seedling transition.

### Identification of TFs that Bind Germination *EXPA* Promoter Sequences

With the (1) identification of *EXPA* gene expression as a promoter of germination under GA-limiting conditions, and (2) identification of 7 *EXPA* genes being induced in *Arabidopsis* embryos during seed germination, we sought to identify the molecular factors that regulate the expression of these germination-promoting genes.

**Figure 2:**
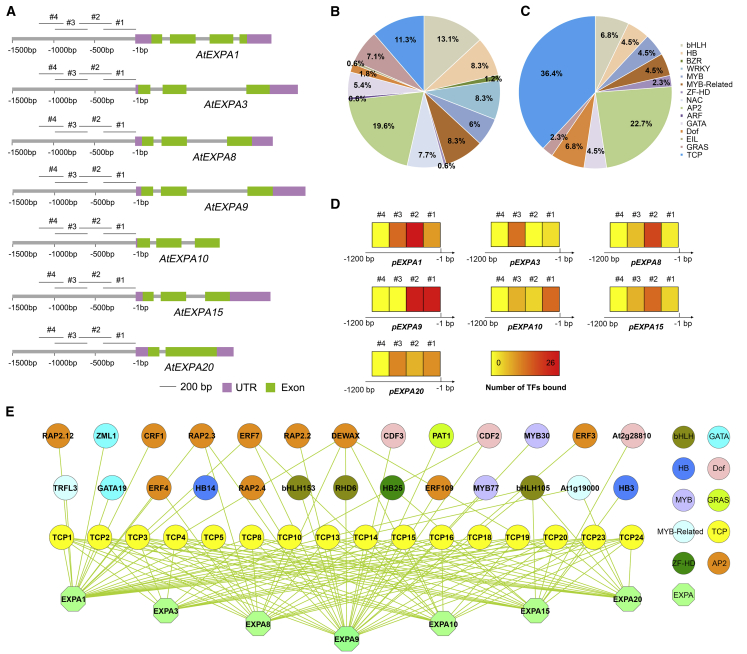
Identification of *EXPA*-Binding Transcription Factors (A) Schematics of genomic regions of embryo-induced *EXPA* genes. 3′ UTR regions are colored in purple, and exons are colored in green. The numbers below the gray lines indicate relative positions upstream of the transcription start site (TSS). Promoter bait fragments used in the Y1h assay are labeled with the numbers 1 to 4, consisting of ~300- to ~400-bp sequences with 100-bp overlaps between adjacent fragments. (B) Pie chart showing the categorization of transcription factor (TF) families included in the Y1h library screened (Data S1B). (C) Pie chart showing the percentage of TFs by family that bound to *EXPA* promoters in yeast (Data S1C). (D) Heatmaps showing the number of TFs that bound to the overlapping promoter fragments of *EXPA* genes. (E) Interaction between TFs and *EXPA* promoter fragments in Y1h assays. Node color indicates TF family after the legend on the right. See also Figures S2–S4.

To identify TFs that bind the 7 *EXPA* promoter sequences, a yeast-1-hybrid (Y1h) assay was used. To generate the *EXPA* promoter bait constructs, 1.2-kb sequence fragments upstream of the transcription start site were cloned and divided into four bait fragments of 300~400 bp with 100-bp-overlapping regions (Figure 2A).

To obtain a suitable TF library for screening, TFs expressed at 7 h after imbibition (HAI) and 12 HAI in the germinating embryo axis, according to publicly available expression data, were selected for subsequent analyses [25] (Data S1A). The selection of the respective time window was guided by the temporal induction of *EXPA* gene expression, reaching high levels by 12 HAI (Figure 1A). A total of 168 of the 255 (66%) seed-expressed TFs were covered in a prey library (Data S1B) [32], representing transcripts from 15 TF families (Figure 2B).

**Figure 3:**
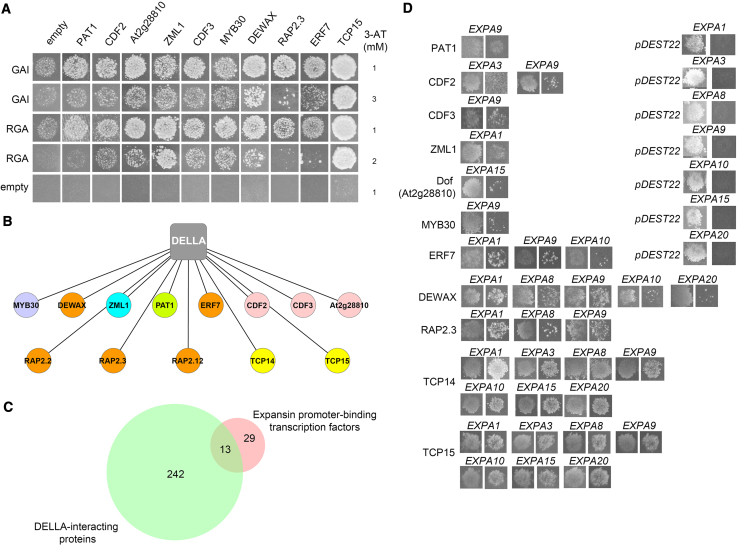
Interaction between *EXPA*-Binding Transcription Factors and DELLA Molecular interactions between TFs and DELLA, and TFs with *EXPA* promoter sequences. (A) Interaction between TFs and DELLA proteins in Y2h assays. (B) Network illustrating the interaction between DELLA and *EXPA* promoter-binding TFs. (C) Venn diagram showing the overlap between DELLA-interacting proteins and TFs that bind the promoter sequences of *EXPA* genes. (D) Y1h colonies showing interaction between TFs that bind to *EXPA* promoter fragments and also interact with DELLA. Left: yeast growth on standard deviation (SD)-Leu-Trp media. Right: yeast growth on SD-Leu-Trp-His + 3-amino-1,2,4-triazole (3-AT). See also Data S2.

Screening of the 28 genomic bait fragments representing the *EXPA* promoter sequences against 168 prey TFs in the Y1h assay resulted in a total of 134 TF-promoter interactions between 42 TFs and genomic regions of 7 *EXPA*s (Figures 2C–2E, 3D, and S2–S4).

Extensive redundancy in the binding of germination-associated *EXPA* promoters was identified as a result of this Y1h screening (Figure 2E; Data S2). A total of 23 of the 42 TFs bound to multiple promoter fragments (55%), whereas each *EXPA* promoter had multiple TFs binding their promoters. Biases in the TF families binding to *EXPA* promoter sequences were also observed, and the TCP and AP2 TF families were enriched (Figure 2C).

### *EXPA* Promoter-Binding TFs Also Interact with DELLA Proteins

Previous work investigating gene expression in seeds has demonstrated the control of GA-promoted germination, and *EXPA* gene expression is regulated by DELLA proteins [18, 33]. To exert their function, DELLA proteins physically interact with TFs such that they sequester their capacity to bind target DNA sequences [33, 34]. We sought to establish whether the TFs identified as *EXPA* promoter-binding proteins also interact with DELLA proteins.

Yeast-2-hybrid (Y2h) screening using truncated (M5) versions of the DELLA proteins REPRESSOR-OF-*ga1-3* (RGA) and GIBBERELLIC ACID INSENSITIVE (GAI) [19, 35] identified multiple TF-DELLA interactions including that of CYCLING DOF FACTOR 2 (CDF2) and CDF3, DECREASE WAX BIOSYNTHESIS (DEWAX), PHYTOCHROME A SIGNAL TRANSDUCTION 1 (PAT1), Dof-type zinc finger (Dof, At2g28810), ZIM-LIKE 1 (ZML1/GATA24), MYB DOMAIN PROTEIN 30 (MYB30), ETHYLENE RESPONSE FACTOR 7 (ERF7), RELATED TO AP2 3 (RAP2.3), and TEOSINTE BRANCHED1/CYCLOIDEA/PCF 15 (TCP15) (Figures 3A and 3B). Several other *EXPA* promoter-binding TFs had been previously reported to interact with DELLA proteins including TCP14 [36] and RAP2.2/RAP2.12 [37]. Of the 42 TFs identified that bind *EXPA* promoters, 13 also physically interact with DELLA proteins (Figure 3C).

### A Molecular Interaction Network Underpinning Seed Germination

**Figure 4:**
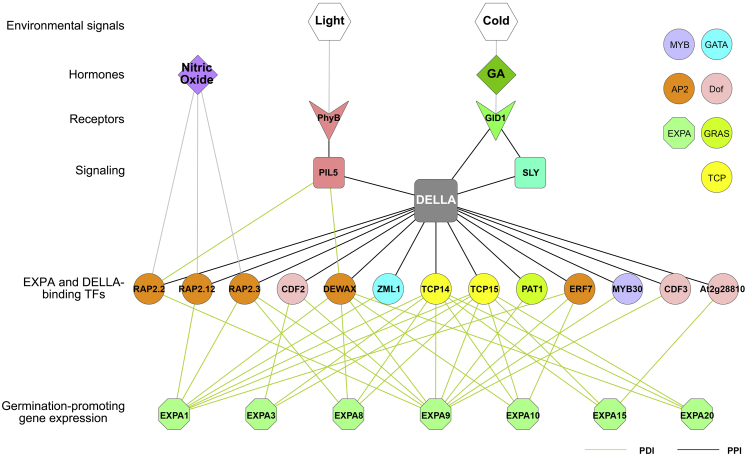
Signal Integration and Growth Regulatory Network in *Arabidopsis* Seeds Molecular regulatory network linking the perception of environmental and hormonal cues to downstream gene expression driving cellular biomechanical changes and embryo growth. TFs are shown as circles, *EXPA* nodes as green octagons, receptors as arrowheads, hormones as squares, and environmental inputs as hexagons. Green edges indicate protein-DNA interactions, black edges indicate protein-protein interactions, and gray edges indicate the regulatory relationships between nodes. Node color indicates TF family after the legend on the right.

Results of the Y1h screen identified TFs that bind to germination-associated *EXPA* genes. These TFs were compared with the Y2h screen identifying DELLA-interacting TFs. This resulted in a network consisting of 134 protein-DNA interactions involving 42 TFs, 7 *EXPA*s, and 13 DELLA-TF protein-protein interactions (Data S2). The subset of this molecular interaction network providing direct links between the perception of GA and the binding of *EXPA* promoter fragments is presented in Figure 4.

The ERFVII family of TFs including RAP2.2, RAP2.3, and RAP2.12 acts as sensors of nitric oxide (NO) in plants [38]. The Y1h screen identifies them as also binding the promoters of *EXPA* genes, providing a potential link between this signaling molecule and the biomechanical modulation of plant growth (Figure 4).

The TF PHYTOCHROME INTERACTING FACTOR3 LIKE5 (PIL5) mediates light-regulated control of seed germination, and interacts with the red light receptor PHYTOCHROME [39]. PIL5 also physically interacts with DELLA proteins [40] and binds to their promoter sequences [41]. Despite the central role of this TF in the control of germination, *EXPA* genes upregulated during germination are not direct targets of PIL5 [42]. The TFs ERF4, CRF1, DEWAX, and RAP2.2 are, however, direct transcriptional targets of PIL5 and bind *EXPA* promoter sequences in the Y1h assay. Both DEWAX and RAP2.2 also interact with DELLA, providing a link between the perception of light and the binding of *EXPA* promoter sequences.

The organization of this molecular interaction network reveals a hierarchical structure of signal integration underpinning the control of seed germination (Figure 4) [4]. The interactions between these molecular agents identify the paths of environmental and hormonal signal perception, their integration, and ultimately the promotion of embryo cell growth through the induction of *EXPA* gene expression.

### *EXPA* Promoter-Binding Proteins Impact GA-Mediated Germination

Whether the TFs identified through molecular interaction screening play a role in GA-mediated *Arabidopsis* seed germination through their control of *EXPA* gene expression was investigated. A total of 13 TFs was screened that both interact with DELLA proteins and bind to the *EXPA* promoter sequences (Figure S5).

**Figure 5:**
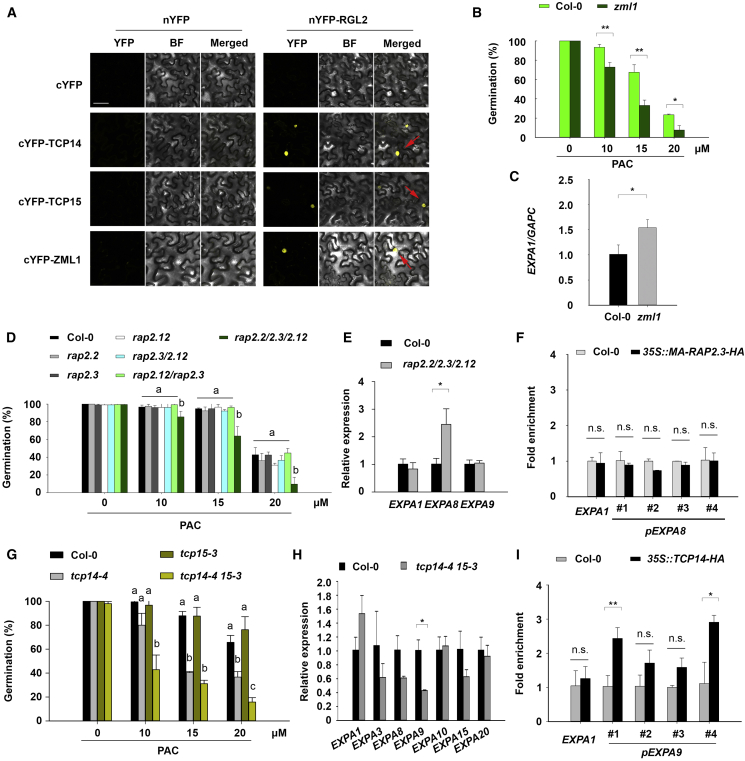
Functional Validation of the Signal Integration Network (A) BiFC assay investigating the interaction between DELLA and TFs in plant cells. Fusion proteins were co-expressed in tobacco leaves by using *Agrobacterium* infiltration and images represent co-transfected cells with visible fluorescence. YFP, fluorescence of yellow fluorescent protein; BF, bright field; merged, merger of the YFP and bright-field images. Red arrows indicate the position of YFP signal. The scale bar indicates 50 μm. (B) Germination of *zml1* imbibed in different concentrations of PAC. (C) Relative expression of the ZML1 target *EXPA1* in the *zml1* background determined by using qPCR. (D) Same as (B) using combinations of *rap2.2 rap2.3* and *rap2.12* mutant seeds. (E) Relative expression of the ERFVII TF targets *EXPA1*, *EXPA8*, and *EXPA9* in the *rap2.2 rap2.3 rap2.12* mutant background determined by qPCR. (F) ChIP using HA-tagged RAP2.3 on the *EXPA8* promoter. The *EXPA1*-coding region was used as a negative control and no specific signals were observed in the negative-control region. (G) Same as (B) using *tcp14* and *tcp15* mutant seeds. (H) qPCR analysis of *EXPA* genes targeted by TCP14 and TCP15. (I) ChIP using HA-tagged TCP14 on the *EXPA9* promoter. The *EXPA1*-coding region is included as a control. Data in (B), (C), (E), (F), (H), and (I) were statistically analyzed by using Student’s *t* test (^∗^p < 0.05, ^∗∗^p < 0.01). Statistically significant differences in (D) and (G) are denoted with different lowercase letters (one-way ANOVA with Tukey post hoc test, p < 0.05). Error bars represent SD from three independent biological repeats. See also Figure S5.

Mutant seeds were initially screened on the GA-synthesis inhibitor PAC, identifying potential GA-mediated germination phenotypes for *TCP14/15*, *ZML1*, and *RAP2.2/2.3/2.12* on 10 μM PAC (Figures S5E–S5M). Before proceeding with further functional analysis of these genes, the interactions between these TFs and DELLA were investigated *in planta*. Although each TCP14/15 and ERFVII TF has been previously reported to interact with DELLAs [36, 37, 40], this has not been established for ZML1. Using bimolecular fluorescence complementation (BiFC) in tobacco leaves, we confirmed the interaction between ZML1 and RGL2 within plant cells (Figure 5A).

Phenotyping mutant seeds on a broader range of PAC concentrations confirmed GA-mediated germination phenotypes for *zml1* (Figure 5B). The regulatory relationship between ZML1 and its putative target *EXPA1* was examined by looking at *EXPA* transcript abundance in the *zml1*-null mutant background. This showed *EXPA1* transcript abundance to be significantly higher than in the wild type (Figure 5C). The inability to recover a transgenic line harboring a detectable epitope-tagged version of this protein prevented the further study of this interaction.

Compared with the wild type, the *rap2.2/2.3/2.12* triple-mutant background showed reduced germination in the presence of PAC, suggesting these ERFVII proteins promote embryo growth in response to GA (Figure 5D). Of the *EXPA* targets of these TFs, the transcript abundance of *EXPA8* was significantly higher in the triple mutant than in the wild type (Figure 5E). Chromatin immunoprecipitation (ChIP) analysis using *35S::MA-RAP2.3-HA* (hemagglutinin), however, did not identify the enrichment of this protein on its putative *EXPA8* promoter fragment targets (Figure 5F). The expression level of *EXPA8* might therefore be indirectly regulated by these *ERFVII* genes.

The *tcp14-4* single and *tcp14-4 15-3* double mutants showed reduced germination in the presence of PAC (Figure 5G). A role for *TCP14* and *TCP15* in GA-stimulated seed germination has been reported previously [43], and was proposed to occur through the regulation of the cell cycle by TCPs within the cells of the radicle. Although the overall contribution of cell division in the radicle to overall embryo growth and germination remains unclear [5, 6], the putative control of *EXPA*s by these TCP TFs was examined as a putative explanation for this reduced germination phenotype.

In the *tcp14-4 15-3* double mutant, the transcript abundance of *EXPA9* was significantly decreased, whereas the expression of the other 6 putative *EXPA* targets was not altered (Figure 5H). ChIP using a *35S::TCP14-HA* construct identified an enrichment of this protein on the promoter of *EXPA9* (Figure 5I). These results collectively suggest TCP14 to be a direct positive regulator of *EXPA9* expression.

By taking together (1) *EXPA* expression is capable of stimulating GA-mediated seed germination (Figure 1C), (2) the *tcp14* mutant shows a reduced capacity to germinate under GA-limited conditions (Figure 5G), and (3) *EXPA9* gene expression in the absence of *TCP14* and *TCP15* (Figure 5H), we sought to determine whether the expression of *EXPA9* is sufficient to account for the reduced germination phenotype observed in the *tcp14-4 tcp15-3* mutant.

**Figure 6:**
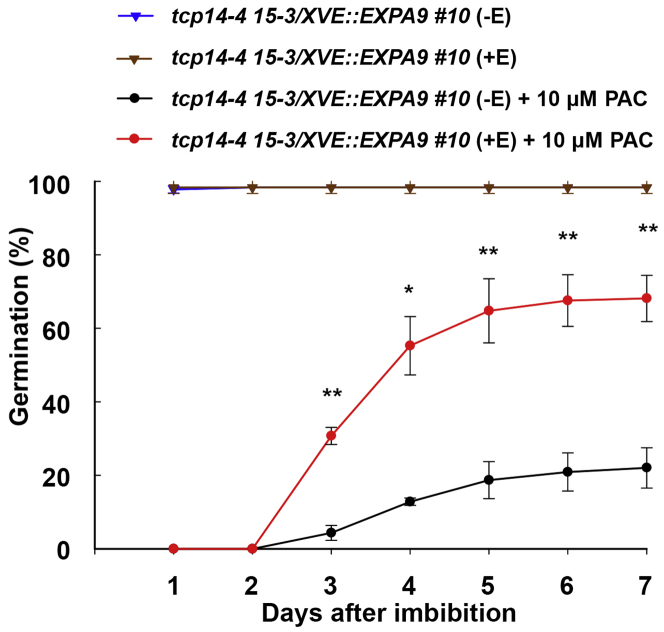
Functional Complementation of TCP Germination by *EXPA* Expression Impact of ectopic expression of *EXPA9* on seed germination upon 10 μM PAC treatment in the *tcp14-4 tcp15-3* mutant background. Data were statistically analyzed by using Student’s *t* test (^∗^p < 0.05, ^∗∗^p < 0.01). See also Figure S6.

To test this, the estrogen-inducible *XVE::EXPA9* construct was introduced into the *tcp14-4 tcp15-3* loss-of-function mutant. Although no significant difference in germination was observed under standard germination conditions, induced expression of *EXPA9* in *tcp14-4 tcp15-3* led to an enhanced germination response in the presence of PAC (Figures 6 and S6). The ectopic expression of *EXPA9* is therefore capable of partially rescuing GA-mediated germination defects in *tcp14-4 tcp15-3*, while partly explaining the mechanistic basis for this phenotype.

### A Resource for Plant Growth and Signal Integration

This study identified 42 TFs capable of binding *EXPA* promoter sequences, 13 of which also interact with DELLA proteins. These TFs represent putative regulators of *EXPA* gene expression, and therefore cell wall biomechanics [10]. The identification of these TFs as direct modulators of plant cell growth provides a focused resource awaiting further characterization. This resource might be used to identify further mechanistic interactions underpinning the modulation of plant growth by linking genetic pathways to the biomechanical changes driving cell expansion. In addition to the provided network file (Data S2), these interactions from this publication have been submitted to the IMEx Consortium (http://www.imexconsortium.org) through IntAct [44] and assigned the identifier IM-27910.

## Discussion

The regulation of plant development is highly plastic in response to the environment [1]. In this study, we reveal the genetic complexity that underpins signal integration in the control of embryo growth during seed germination. These insights reveal the extent and nature of the redundancy that underlies the control of the downstream gene expression promoting GA-mediated embryo growth. This is observed in terms of the number of *EXPA* genes that are induced during germination, and the large number of TFs that bind to the promoters of these genes (Figure 2E). This network further reveals the paths of information flow from the perception of environmental signals to the downstream gene expression that drives the seed-to-seedling developmental transition (Figure 4).

Although a variety of environmental, hormonal, and genetic factors have been described to regulate seed germination [3], how these signals are integrated and transduced into embryo growth remains unclear. This study addressed this gap by performing targeted molecular interaction mapping to establish a network underpinning signal integration and seed-germination-driving gene expression in *Arabidopsis* (Figure 4). The network linked the perception of environmental (light quality) and hormonal (GA and NO) signals to the gene expression responsible for the biomechanical changes driving the seed-to-seedling transition (*EXPA*).

Although *EXPA* gene expression was shown to promote GA-mediated embryo growth (Figure 1C), the regulation of these downstream targets is highly redundant, and multiple TFs bound to their associated upstream promoter fragments (Figure 2E). This redundancy in the integration of signals into *EXPA* gene expression likely provides robustness to the germination process, whereby a single TF-*EXPA* interaction rarely impacts the seed-to-seedling transition (Figure S5). This redundancy could explain the relatively small number of GA-related germination phenotypes identified in seeds carrying mutations in single genes or restricted to single gene families. Despite this redundancy, phenotypes in null mutants of the ERFVII (RAP/2.2/2.3/2.12), TCP14/15, and ZML1 TFs were identified (Figures 5B, 5D, and 5G).

The *tcp14 tcp15* mutant showed reduced germination in GA-limited conditions (Figure 5G), and TCP14 directly bound and promoted *EXPA9* expression (Figures 5H and 5I). A GA-mediated germination phenotype for TCP14 and TCP15 has been proposed previously to act through the control of the cell cycle in the radicle [43]. This work extends the role of these genes to the promotion of cell expansion through the control of *EXPA9* gene expression, a proposal consistent with the description of this TF as a promoter of embryo growth potential [45]. This finding further suggests the mechanistic basis of reduced germination in the *tcp14 tcp15* mutant background to be at least partially due to a reduction in *EXPA* expression, as supported by the partial rescue of this phenotype under GA-limiting conditions (Figure 6).

The uncovering of this network provides insight into a fundamental gap in our understanding of a complex biological process: how multiple environmental inputs are integrated to create a single developmental output, in this instance, how environmental signals are used to regulate the gene expression altering cellular biomechanics and embryo growth in seeds. It further highlights the importance of understanding the complexity of transcriptional regulation as a whole system, while providing a resource for further exploitation by understanding how the activity of the TFs that bind to *EXPA*s modulates plant growth.

The identification of downstream targets of developmental processes enables the mechanistic basis of phenotypes to be established and the comprehensive mapping of the molecular interactions underpinning developmental phase transitions. Similar approaches and the resources generated by these studies represent powerful ways to understand plant development.

## STAR★Methods

### Key Resources Table

**Table:** 

REAGENT or RESOURCE	SOURCE	IDENTIFIER
**Antibodies**		

Anti-HA tag antibody - ChIP Grade	Abcam	Cat#ab9110
Goat Anti-Mouse IgG-Peroxidase antibody	Sigma-Aldrich	Cat#A4416

**Bacterial and Virus Strains**		

*Escherichia coli* DH5α	[27]	
*Agrobacterium tumefaciens* (strain GV3101)	[27]	

**Chemicals, Peptides, and Recombinant Proteins**		

Phusion DNA Polymerase	NEB	M0530
Murashige & Skoog Medium	Duchefa Biochemie	Cat#M0221
β-estradiol	Sigma-Aldrich	Cat#E2758
3-Amino-1,2,4-triazole	Sigma-Aldrich	Cat#A8056
Taq DNA Polymerase	NEB	Cat#M0273

**Critical Commercial Assays**		

RNeasy PowerPlant Kit	QIAGEN	Cat#13500-50
RNeasy Plant Mini Kit	QIAGEN	Cat#74903
Plant DNeasy Kit	QIAGEN	Cat#69104
Gateway BP Clonase II Enzyme mix	Invitrogen	Cat#11789020
Gateway LR Clonase II Enzyme mix	Invitrogen	Cat#11791020
SuperScript II Reverse Transcriptase	Invitrogen	Cat#18064022
Brilliant II QPCR Master Mix with ROX	Agilent	Cat#600806
Pierce Protein A/G Magnetic Beads	Invitrogen	Cat#88802

**Experimental Models: Cell Lines**		

Yeast strain AH109 (*MATɑ*)	Clontech	N/A
Yeast strain Y187 (*MATɑ*)	Clontech	Cat#630457
Yeast strain Y8800 (*MATɑ*)	Clontech	N/A
Yeast strain Y8930 (*MATɑ*)	Clontech	N/A

**Experimental Models: Organisms/Strains**		

*Arabidopsis thaliana* Col-0	[27]	
*Arabidopsis thaliana At2g28810*	*Arabidopsis* Biological Resource Center	Cat#SALK_056801C
*Arabidopsis thaliana erf7*	*Arabidopsis* Biological Resource Center	Cat#SALK_032229
*Arabidopsis thaliana myb30*	*Arabidopsis* Biological Resource Center	Cat#SALK_027644C
*Arabidopsis thaliana pat1*	*Arabidopsis* Biological Resource Center	Cat#SALK_064220C
*Arabidopsis thaliana TCP14 TPTD*	*Arabidopsis* Biological Resource Center	Cat#TPT_3.47620.1D
*Arabidopsis thaliana TCP15 TPTC*	*Arabidopsis* Biological Resource Center	Cat#TPT_1.69690.1C
*Arabidopsis thaliana RAP2.3 TPTD*	*Arabidopsis* Biological Resource Center	Cat#TPT_3.16770.1D
*Arabidopsis thaliana* Col-0 *XVE::EXPA9*	This study	N/A
*Arabidopsis thaliana tcp14-4 15-3 XVE:EXPA9*	This study	N/A
*Arabidopsis thaliana tcp14-4 35S::TCP14-HA*	This study	N/A
*Arabidopsis thaliana cdf2-1*	[46]	N/A
*Arabidopsis thaliana cdf3-1*	[46]	N/A
*Arabidopsis thaliana cdf2-13-1*	[46]	N/A
*Arabidopsis thaliana CDF2-OX*	[47]	N/A
*Arabidopsis thaliana dewax*	[48]	N/A
*Arabidopsis thaliana iDEWAX*	[48]	N/A
*Arabidopsis thaliana zml1*	[49]	N/A
*Arabidopsis thaliana rap2.2*	[50]	N/A
*Arabidopsis thaliana ebp*	[50]	N/A
*Arabidopsis thaliana rap2.12*	[50]	N/A
*Arabidopsis thaliana rap2.2 2.12*	[50]	N/A
*Arabidopsis thaliana rap2.12 ebp*	[50]	N/A
*Arabidopsis thaliana rap2.2 2.3 2.12*	[50]	N/A
*Arabidopsis thaliana tcp14-4*	[51]	N/A
*Arabidopsis thaliana tcp15-3*	[51]	N/A
*Arabidopsis thaliana tcp14-4 15-3*	[51]	N/A

**Oligonucleotides**		

Primers	Table S1	N/A

**Recombinant DNA**		

*pHISLEU2GW*	[32]	N/A
*pHISLEU2GW-pEXPA1*	This study	N/A
*pHISLEU2GW-pEXPA3*	This study	N/A
*pHISLEU2GW-pEXPA8*	This study	N/A
*pHISLEU2GW-pEXPA9*	This study	N/A
*pHISLEU2GW-pEXPA10*	This study	N/A
*pHISLEU2GW-pEXPA15*	This study	N/A
*pHISLEU2GW-pEXPA20*	This study	N/A
*pDEST-AD*	Invitrogen	N/A
*pDEST-DB*	Invitrogen	N/A
*pDEST-DB-RGAΔN*	This study	N/A
*pDEST-DB-GAIΔN*	This study	N/A
*pSPYNE*	[52]	N/A
*pSPYNE-RGL2*	This study	N/A
*pSPYCE*	[52]	N/A
*pSPYCE-TCP14*	This study	N/A
*pSPYCE-TCP15*	This study	N/A
*pSPYCE-ZML1*	This study	N/A
*pGWB14*	RIKEN BRC	N/A
*pGWB14-TCP14*	This study	N/A
*pER8-GW*	[30]	N/A
*pER8-GW-EXPA9*	This study	N/A

**Software and Algorithms**		

CytoScape	[53]	https://cytoscape.org/
matrix2png	[54]	https://matrix2png.msl.ubc.ca/index.html

### Resource Availability

#### Lead Contact

Further information and requests for resources should be directed to and will be fulfilled by the Lead Contact, George W. Bassel (george.bassel@warwick.ac.uk).

#### Materials Availability

This study did not generate new unique reagents.

#### Data and Code Availability

All datasets generated or analyzed during this study are included in the manuscript. The interactions from this publication have been submitted to the IMEx (http://www.imexconsortium.org) consortium through IntAct [44] and assigned the identifier IM-27910.

### Experimental Model and Subject Details

#### Plant Material and growth conditions

Seeds were grown on half-strength Murashige and Skoog (MS) medium with 0.8% Agar under 16h/8h light/dark cycles. 2-week-old seedlings were transferred to soil and grown in a greenhouse. Freshly harvested seeds were stored at room temperature for 1-2 months.

*Arabidopsis* T-DNA insertion lines were obtained from *Arabidopsis* Biological Resource Center (ABRC): *At2g28810* mutant (SALK_056801C), *erf7* (SALK_032229), *myb30* (SALK_027644C), *pat1* (SALK_064220C). Following lines were kindly provided by authors and have been previously described: *cdf2-1*, *cdf3-1*, *cdf2-13-1*, and *CDF2-OX* [46, 47]; *dewax* and *iDEWAX* [48]; *zml1* [49]; *rap2.2*, *ebp*, *rap2.12*, *rap2.2 2.12*, *rap2.12 ebp*, and *rap2.2 2.3 2.12* [50]; *tcp14-4*, *tcp15-3*, and *tcp14-4 15-3* [51].

### Method Details

#### Plasmid construction and plant transformation

To generate the estrogen-inducible *XVE::EXPA9* construct, full-length cDNA coding sequence of *EXPA9* was amplified using primers described in Table S1 with Phusion DNA Polymerase (NEB, M0530), and recombined in *pDONRzeo* using BP clonase II (Invitrogen). The fragment was subsequently transferred from the entry vector into the estrogen inducible *pER8GW* [30] by LR clonase reaction (Invitrogen).

For the *35S::TCP14-HA* construct, the coding sequence without the stop codon of *TCP14* was amplified and inserted into *pDONRzeo* by BP reaction, then recombined with *pGWB14* by LR reaction (Invitrogen).

The resulting plasmids were transformed into *Agrobacterium tumefaciens* strain GV3101 and introduced into *Arabidopsis thaliana* by the floral dipping method [55]. Transgenic progeny seeds were selected on half-strength MS medium with 20 mg/L hygromycin [55].

#### Phenotypic analyses

Germination assays were performed by surface sterilizing seeds and pipetting them onto 1/2 MS medium supplemented with PAC as required to achieve specific concentrations. For estrogen inducible lines, seeds were either treated with 30 μM β-estradiol or ethanol (mock). After sowing, seeds were kept at 4°C under dark conditions for 3 days, and then transferred to a controlled growth chamber under 16h/8h light-dark cycles at 22°C.

#### Gene expression analyses

Total RNA was isolated either from *Arabidopsis* seeds using the RNeasy PowerPlant Kit or from seedlings using RNeasy Plant Mini Kit (QIAGEN) according to the manufacturer’s instructions. DNA in RNA samples was removed with DNase I (Thermo Fisher Scientific) and RNA was reverse-transcribed into cDNA using SuperScript II Reverse Transcriptase (Invitrogen). Quantitative PCR was performed in 96-well blocks with Brilliant II QPCR Master Mix with ROX (Agilent, #600806) on the AriaMx Real-Time PCR system. Gene expression was normalized using internal control *GLYCERALDEHYDE-3-PHOSPHATE DEHYDROGENASE C SUBUNIT* (*GAPC*) (*At3g04120*) [56]. RT-PCR was performed with Taq DNA Polymerase (NEB, #M0273) on a thermal cycler. Analysis of *EXPA9* were subjected to amplication for 26 and 30 cycles, and analysis of *GAPC* was followed by 26 cycles.

#### Yeast one hybrid screening of *Arabidopsis* cDNA libraries

TFs included in the Y1h screening library were determined by examining those expressed in the germinating embryo at 7 HAI and 12 HAI [25], prior to or concurrent with the induction of *EXPA* gene expression. 168 embryo expressed TFs were available in the REGIA + REGULATORS RR Library [32], in which TF cDNAs fused to an N-terminal GAL4-activation domain in *pDEST22* (Invitrogen). Prey clones were transformed into yeast strain AH109 (MATɑ, Clontech) according to manufacturer’s instructions.

Y1h screening was performed as described previously [57]. Promoter sequences for *EXPA1* (*At1g69530*), *EXPA3* (*At2g37640*), *EXPA8* (*At2g40610*), *EXPA9* (*At5g02260*), *EXPA10* (*At1g26770*), *EXPA15* (*At2g03090*), and *EXPA20* (*At4g38210*) consisting of 300 ~400 bp fragments with 100 bp overlaps were cloned into *pDONRZeo*, and then into the destination vector *pHISLEU2GW*. Bait vectors were then transformed into yeast strain Y187 (MATɑ, Clontech) and tested for autoactivation. Several fragments required the addition of 3-amino-1,2,4-triazol (3-AT) to enable selection of interacting proteins. Mating was performed by mixing 3 μL of each bait and prey onto YPDA plates, with subsequent replica-plating (after 24 h) onto SD-Leu-Trp and SD-Leu-Trp-His (+ 3-AT where required) and grown for two days to confirm mating and provide selection for interaction. All positive interactions in yeast were re-tested and sequenced.

#### Protein-protein interaction assays

Protein interaction screening in yeast was performed using N-terminal truncations of RGA and GAI. These were cloned into *pDEST-DB* and screened by yeast mating against *EXPA* promoter-binding TFs fused to a GAL4 activation domain (AD) expressed via *pDEST-AD* [58] following previously described protocols [59]. To detect AD auto-activators, the AD-fused transcription factor collection in MATɑ Y8800 yeast strains was mated with the empty *pDEST-DB* expressing MATɑ Y8930, then selected using 1 mM 3-amino-1,2,4-triazole (3-AT). Protein interaction screens were repeated twice with 1 mM 3-AT and a second time with 2 and 3 mM 3-AT for RGA and GAI, respectively.

#### BiFC protein-protein interaction *in planta*

Full-length TF coding sequences and *RGL2* were cloned into *pSPYCE* or *pSPYNE* vectors containing either C- or N-terminal portions of yellow fluorescence protein [52] and transformed into *Agrobacterium* strain GV3101. Bacteria were grown overnight at 28°C in LB medium and resuspended in infiltration buffer (10 mM MES, pH5.7, 10 mM MgCl_2_, and 150 mM acetosyringone). To visualize the fluorescence signal, different combinations of bacteria cells were co-infiltrated into the leaves of 4-week-old *Nicotiana benthamiana*. YFP signals were assayed 3 days after inoculation and excited at 488 nm using a Zeiss LSM510 microscope.

#### Chromatin Immunoprecipitation

ChIP was performed as described previously with minor modifications [60]. Seedlings (6 days after imbibition) grown on half-strength MS medium supplemented with 10 μM GA_3_ were used for CHIP-qPCR analyses. Chromatin was isolated from 2 g of seedlings and sheared by sonication to fragments of 400 bp. Following isolation, sonicated chromatin was precleared with Dynabeads Protein A/G (Invitrogen) and then immunoprecipitated overnight using Anti-HA (Abcam, ab9110) polyclonal antibodies. qPCR analyses were performed using Brilliant II QPCR Master Mix with ROX (Agilent, #600806). Fold enrichment was calculated by normalizing the amount of target fragment first to the internal control gene (*ACTIN2*) and then to the corresponding amount in the wild-type (Col-0). The primers used for ChIP-qPCR are listed in Table S1.

#### Bioinformatics analyses

The network graph was visualized using CytoScape (version 3.7) [53]. Expression profile of EXPAs was generated in the Matrix2png program (https://matrix2png.msl.ubc.ca/index.html) [54].

### Quantification and Statistical Analysis

Statistical analysis was implemented using either EXCEL or SPSS software. Data shown in the figures are representing an average of biological replicates. All seed germination test had at least 50 seeds in each biological replication. Two-tailed Student’s t test was performed with the t.test() function in EXCEL. One-way ANOVAs with post hoc Turkey test was carried out by SPSS (version 16.0) at a significance level of p < 0.05. Details of statistical tests are provided in figure legends.

## Supplemental Information

Document S1. Figures S1–S6

Table S1. List of Primers Used, Related to STAR Methods

Data S1. TFs Expressed in the Germinating Embryo and Included in the Y1h Library, Related to Figure 2 (A) Transcription factors expressed in the embryo during the early stages of seed germination. (B) Transcription factors included in the Y1h library. (C) Transcription factors identified in the GRN.

Data S2. Cytoscape File Containing the Interaction Network Derived in This Study, Related to Figure 3

Document S2. Article plus Supplemental Information

## References

[1] Domagalska M.A., Leyser O. (2011). Signal integration in the control of shoot branching. Nat. Rev. Mol. Cell Biol..

[2] Koornneef M., Bentsink L., Hilhorst H. (2002). Seed dormancy and germination. Curr. Opin. Plant Biol..

[3] Holdsworth M.J., Bentsink L., Soppe W.J. (2008). Molecular networks regulating *Arabidopsis* seed maturation, after-ripening, dormancy and germination. New Phytol..

[4] Bassel G.W. (2016). To grow or not to grow?. Trends Plant Sci..

[5] Sliwinska E., Bassel G.W., Bewley J.D. (2009). Germination of *Arabidopsis thaliana* seeds is not completed as a result of elongation of the radicle but of the adjacent transition zone and lower hypocotyl. J. Exp. Bot..

[6] Bassel G.W., Stamm P., Mosca G., Barbier de Reuille P., Gibbs D.J., Winter R., Janka A., Holdsworth M.J., Smith R.S. (2014). Mechanical constraints imposed by 3D cellular geometry and arrangement modulate growth patterns in the *Arabidopsis* embryo. Proc. Natl. Acad. Sci. USA.

[7] Nakabayashi K., Okamoto M., Koshiba T., Kamiya Y., Nambara E. (2005). Genome-wide profiling of stored mRNA in *Arabidopsis thaliana* seed germination: epigenetic and genetic regulation of transcription in seed. Plant J..

[8] Ogawa M., Hanada A., Yamauchi Y., Kuwahara A., Kamiya Y., Yamaguchi S. (2003). Gibberellin biosynthesis and response during *Arabidopsis* seed germination. Plant Cell.

[9] Narsai R., Gouil Q., Secco D., Srivastava A., Karpievitch Y.V., Liew L.C., Lister R., Lewsey M.G., Whelan J. (2017). Extensive transcriptomic and epigenomic remodelling occurs during *Arabidopsis thaliana* germination. Genome Biol..

[10] Cosgrove D.J. (2000). Loosening of plant cell walls by expansins. Nature.

[11] Chen F., Nonogaki H., Bradford K.J. (2002). A gibberellin-regulated xyloglucan endotransglycosylase gene is expressed in the endosperm cap during tomato seed germination. J. Exp. Bot..

[12] Ren C., Kermode A.R. (2000). An increase in pectin methyl esterase activity accompanies dormancy breakage and germination of yellow cedar seeds. Plant Physiol..

[13] Sitrit Y., Hadfield K.A., Bennett A.B., Bradford K.J., Downie A.B. (1999). Expression of a polygalacturonase associated with tomato seed germination. Plant Physiol..

[14] Sánchez-Montesino R., Bouza-Morcillo L., Marquez J., Ghita M., Duran-Nebreda S., Gómez L., Holdsworth M.J., Bassel G., Oñate-Sánchez L. (2019). A regulatory module controlling GA-mediated endosperm cell expansion is critical for seed germination in *Arabidopsis*. Mol. Plant.

[15] Debeaujon I., Koornneef M. (2000). Gibberellin requirement for *Arabidopsis* seed germination is determined both by testa characteristics and embryonic abscisic acid. Plant Physiol..

[16] Bassel G.W., Mullen R.T., Bewley J.D. (2008). Procera is a putative DELLA mutant in tomato (*Solanum lycopersicum*): effects on the seed and vegetative plant. J. Exp. Bot..

[17] Lee K.P., Piskurewicz U., Turecková V., Strnad M., Lopez-Molina L. (2010). A seed coat bedding assay shows that RGL2-dependent release of abscisic acid by the endosperm controls embryo growth in *Arabidopsis* dormant seeds. Proc. Natl. Acad. Sci. USA.

[18] Lee S., Cheng H., King K.E., Wang W., He Y., Hussain A., Lo J., Harberd N.P., Peng J. (2002). Gibberellin regulates *Arabidopsis* seed germination via RGL2, a GAI/RGA-like gene whose expression is up-regulated following imbibition. Genes Dev..

[19] de Lucas M., Davière J.-M., Rodríguez-Falcón M., Pontin M., Iglesias-Pedraz J.M., Lorrain S., Fankhauser C., Blázquez M.A., Titarenko E., Prat S. (2008). A molecular framework for light and gibberellin control of cell elongation. Nature.

[20] Achard P., Cheng H., De Grauwe L., Decat J., Schoutteten H., Moritz T., Van Der Straeten D., Peng J., Harberd N.P. (2006). Integration of plant responses to environmentally activated phytohormonal signals. Science.

[21] Mukhtar M.S., Carvunis A.-R., Dreze M., Epple P., Steinbrenner J., Moore J., Tasan M., Galli M., Hao T., Nishimura M.T. (2011). Independently evolved virulence effectors converge onto hubs in a plant immune system network. Science.

[22] Taylor-Teeples M., Lin L., de Lucas M., Turco G., Toal T.W., Gaudinier A., Young N.F., Trabucco G.M., Veling M.T., Lamothe R. (2015). An *Arabidopsis* gene regulatory network for secondary cell wall synthesis. Nature.

[23] Gaudinier A., Rodriguez-Medina J., Zhang L., Olson A., Liseron-Monfils C., Bågman A.-M., Foret J., Abbitt S., Tang M., Li B. (2018). Transcriptional regulation of nitrogen-associated metabolism and growth. Nature.

[24] O’Malley R.C., Huang S.-C., Song L., Lewsey M.G., Bartlett A., Nery J.R., Galli M., Gallavotti A., Ecker J.R. (2016). Cistrome and epicistrome features shape the regulatory DNA landscape. Cell.

[25] Dekkers B.J.W., Pearce S., van Bolderen-Veldkamp R.P., Marshall A., Widera P., Gilbert J., Drost H.-G., Bassel G.W., Müller K., King J.R. (2013). Transcriptional dynamics of two seed compartments with opposing roles in *Arabidopsis* seed germination. Plant Physiol..

[26] Linkies A., Müller K., Morris K., Turecková V., Wenk M., Cadman C.S., Corbineau F., Strnad M., Lynn J.R., Finch-Savage W.E., Leubner-Metzger G. (2009). Ethylene interacts with abscisic acid to regulate endosperm rupture during germination: a comparative approach using *Lepidium sativum* and *Arabidopsis thaliana*. Plant Cell.

[27] Stamm P., Topham A.T., Mukhtar N.K., Jackson M.D., Tomé D.F., Beynon J.L., Bassel G.W. (2017). The transcription factor ATHB5 affects GA-mediated plasticity in hypocotyl cell growth during seed germination. Plant Physiol..

[28] Kende H., Bradford K., Brummell D., Cho H.-T., Cosgrove D., Fleming A., Gehring C., Lee Y., McQueen-Mason S., Rose J., Voesenek L.A. (2004). Nomenclature for members of the expansin superfamily of genes and proteins. Plant Mol. Biol..

[29] Graeber K., Linkies A., Steinbrecher T., Mummenhoff K., Tarkowská D., Turečková V., Ignatz M., Sperber K., Voegele A., de Jong H. (2014). DELAY OF GERMINATION 1 mediates a conserved coat-dormancy mechanism for the temperature- and gibberellin-dependent control of seed germination. Proc. Natl. Acad. Sci. USA.

[30] Papdi C., Abrahám E., Joseph M.P., Popescu C., Koncz C., Szabados L. (2008). Functional identification of *Arabidopsis* stress regulatory genes using the controlled cDNA overexpression system. Plant Physiol..

[31] Piskurewicz U., Jikumaru Y., Kinoshita N., Nambara E., Kamiya Y., Lopez-Molina L. (2008). The gibberellic acid signaling repressor RGL2 inhibits *Arabidopsis* seed germination by stimulating abscisic acid synthesis and ABI5 activity. Plant Cell.

[32] Castrillo G., Turck F., Leveugle M., Lecharny A., Carbonero P., Coupland G., Paz-Ares J., Oñate-Sánchez L. (2011). Speeding *cis*-*trans* regulation discovery by phylogenomic analyses coupled with screenings of an arrayed library of *Arabidopsis* transcription factors. PLoS One.

[33] Cao D., Cheng H., Wu W., Soo H.M., Peng J. (2006). Gibberellin mobilizes distinct DELLA-dependent transcriptomes to regulate seed germination and floral development in *Arabidopsis*. Plant Physiol..

[34] Davière J.-M., Achard P. (2013). Gibberellin signaling in plants. Development.

[35] Lantzouni O., Alkofer A., Falter-Braun P., Schwechheimer C. (2020). GROWTH-REGULATING FACTORS interact with DELLAs and regulate growth in cold stress. Plant Cell.

[36] Davière J.-M., Wild M., Regnault T., Baumberger N., Eisler H., Genschik P., Achard P. (2014). Class I TCP-DELLA interactions in inflorescence shoot apex determine plant height. Curr. Biol..

[37] Marín-de la Rosa N., Sotillo B., Miskolczi P., Gibbs D.J., Vicente J., Carbonero P., Oñate-Sánchez L., Holdsworth M.J., Bhalerao R., Alabadí D., Blázquez M.A. (2014). Large-scale identification of gibberellin-related transcription factors defines group VII ETHYLENE RESPONSE FACTORS as functional DELLA partners. Plant Physiol..

[38] Gibbs D.J., Md Isa N., Movahedi M., Lozano-Juste J., Mendiondo G.M., Berckhan S., Marín-de la Rosa N., Vicente Conde J., Sousa Correia C., Pearce S.P. (2014). Nitric oxide sensing in plants is mediated by proteolytic control of group VII ERF transcription factors. Mol. Cell.

[39] Oh E., Kim J., Park E., Kim J.-I., Kang C., Choi G. (2004). PIL5, a phytochrome-interacting basic helix-loop-helix protein, is a key negative regulator of seed germination in *Arabidopsis thaliana*. Plant Cell.

[40] Gallego-Bartolomé J., Minguet E.G., Marín J.A., Prat S., Blázquez M.A., Alabadí D. (2010). Transcriptional diversification and functional conservation between DELLA proteins in *Arabidopsis*. Mol. Biol. Evol..

[41] Oh E., Yamaguchi S., Hu J., Yusuke J., Jung B., Paik I., Lee H.-S., Sun T.P., Kamiya Y., Choi G. (2007). PIL5, a phytochrome-interacting bHLH protein, regulates gibberellin responsiveness by binding directly to the GAI and RGA promoters in *Arabidopsis* seeds. Plant Cell.

[42] Oh E., Kang H., Yamaguchi S., Park J., Lee D., Kamiya Y., Choi G. (2009). Genome-wide analysis of genes targeted by PHYTOCHROME INTERACTING FACTOR 3-LIKE5 during seed germination in *Arabidopsis*. Plant Cell.

[43] Resentini F., Felipo-Benavent A., Colombo L., Blázquez M.A., Alabadí D., Masiero S. (2015). TCP14 and TCP15 mediate the promotion of seed germination by gibberellins in *Arabidopsis thaliana*. Mol. Plant.

[44] Orchard S., Ammari M., Aranda B., Breuza L., Briganti L., Broackes-Carter F., Campbell N.H., Chavali G., Chen C., del-Toro N. (2014). The MIntAct project—IntAct as a common curation platform for 11 molecular interaction databases. Nucleic Acids Res..

[45] Tatematsu K., Nakabayashi K., Kamiya Y., Nambara E. (2008). Transcription factor AtTCP14 regulates embryonic growth potential during seed germination in *Arabidopsis thaliana*. Plant J..

[46] Fornara F., Panigrahi K.C.S., Gissot L., Sauerbrunn N., Rühl M., Jarillo J.A., Coupland G. (2009). *Arabidopsis* DOF transcription factors act redundantly to reduce CONSTANS expression and are essential for a photoperiodic flowering response. Dev. Cell.

[47] Sun Z., Guo T., Liu Y., Liu Q., Fang Y. (2015). The roles of *Arabidopsis* CDF2 in transcriptional and posttranscriptional regulation of primary microRNAs. PLoS Genet..

[48] Go Y.S., Kim H., Kim H.J., Suh M.C. (2014). *Arabidopsis* cuticular wax biosynthesis is negatively regulated by the DEWAX gene encoding an AP2/ERF-type transcription factor. Plant Cell.

[49] Shaikhali J., de Dios Barajas-Lopéz J., Ötvös K., Kremnev D., Garcia A.S., Srivastava V., Wingsle G., Bako L., Strand Å. (2012). The CRYPTOCHROME1-dependent response to excess light is mediated through the transcriptional activators ZINC FINGER PROTEIN EXPRESSED IN INFLORESCENCE MERISTEM LIKE1 and ZML2 in *Arabidopsis*. Plant Cell.

[50] Abbas M., Berckhan S., Rooney D.J., Gibbs D.J., Vicente Conde J., Sousa Correia C., Bassel G.W., Marín-de la Rosa N., León J., Alabadí D. (2015). Oxygen sensing coordinates photomorphogenesis to facilitate seedling survival. Curr. Biol..

[51] Kieffer M., Master V., Waites R., Davies B. (2011). TCP14 and TCP15 affect internode length and leaf shape in *Arabidopsis*. Plant J..

[52] Walter M., Chaban C., Schütze K., Batistic O., Weckermann K., Näke C., Blazevic D., Grefen C., Schumacher K., Oecking C. (2004). Visualization of protein interactions in living plant cells using bimolecular fluorescence complementation. Plant J..

[53] Shannon P., Markiel A., Ozier O., Baliga N.S., Wang J.T., Ramage D., Amin N., Schwikowski B., Ideker T. (2003). Cytoscape: a software environment for integrated models of biomolecular interaction networks. Genome Res..

[54] Pavlidis P., Noble W.S. (2003). Matrix2png: a utility for visualizing matrix data. Bioinformatics.

[55] Zhang X., Henriques R., Lin S.-S., Niu Q.-W., Chua N.-H. (2006). Agrobacterium-mediated transformation of Arabidopsis thaliana using the floral dip method. Nat Protoc.

[56] Dill A., Thomas S.G., Hu J., Steber C.M., Sun T.P. (2004). The *Arabidopsis* F-box protein SLEEPY1 targets gibberellin signaling repressors for gibberellin-induced degradation. Plant Cell.

[57] Hickman R., Hill C., Penfold C.A., Breeze E., Bowden L., Moore J.D., Zhang P., Jackson A., Cooke E., Bewicke-Copley F. (2013). A local regulatory network around three NAC transcription factors in stress responses and senescence in *Arabidopsis* leaves. Plant J..

[58] Pruneda-Paz J.L., Breton G., Nagel D.H., Kang S.E., Bonaldi K., Doherty C.J., Ravelo S., Galli M., Ecker J.R., Kay S.A. (2014). A genome-scale resource for the functional characterization of *Arabidopsis* transcription factors. Cell Rep..

[59] Altmann M., Altmann S., Falter C., Falter-Braun P. (2018). High-quality yeast-2-hybrid interaction network mapping. Curr. Protoc. Plant Biol..

[60] Gendrel A.-V., Lippman Z., Martienssen R., Colot V. (2005). Profiling histone modification patterns in plants using genomic tiling microarrays. Nat. Methods.

