# Supplementary material for: A Molecular Signal Integration Network Underpinning Arabidopsis Seed Germination
Source: Curr Biol. 2020 Oct 5;30(19):3703–3712.e4. doi: 10.1016/j.cub.2020.07.012 (PMC7544511; doi:10.1016/j.cub.2020.07.012)
Supplement: Document S1. Figures S1–S6 [file mmc1.pdf]

Current Biology, Volume *30*

Supplemental Information

A Molecular Signal Integration Network
Underpinning *Arabidopsis* Seed Germination

Hao Xu, Ourania Lantzouni, Tonko Bruggink, Rene Benjamins, Frank Lanfermeijer, Katherine Denby, Claus Schwechheimer, and George W. Bassel

Supplementary Information

A molecular signal integration network underpinning *Arabidopsis* seed germination

Hao Xu[1], Ourania Lantzouni[2], Tonko Bruggink[4], Rene Benjamins[4], Frank Lanfermeijer[4], Katherine Denby[3], Claus Schwechheimer[2], George W. Bassel[1,*]

[1] School of Life Sciences, University of Warwick, Coventry, CV4 7AL, UK
[2] Plant Systems Biology, Technical University of Munich, 85354 Freising, Germany
[3] Department of Biology, University of York, York, YO10 5DD, UK
[4] Applied Physiology, Syngenta Seeds B.V., P.O. Box 2, NL-1600AA Enkhuizen, The Netherlands

CORRESPONDING AUTHOR

[*]To whom correspondence should be addressed: george.bassel@warwick.ac.uk (G.W.B);

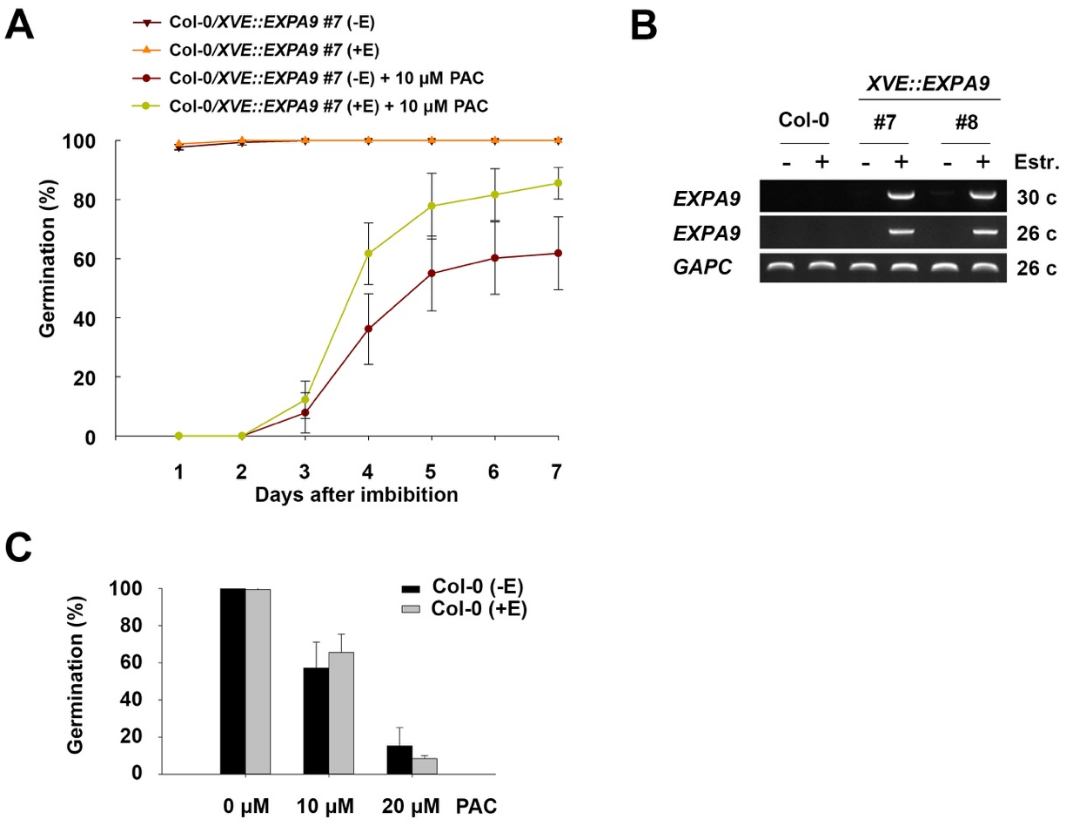

Figure S1. Impact of *EXPA* expression on germination. Related to Figure 1. (A) Germination of *XVE::EXPA9* line 7 seeds on water and 10 µM PAC with and without the ectopic induction using estrogen (+/- E). (B) Induction of *EXPA9* in *XVE::EXPA9* lines 7 and 8 confirmed by RT-PCR using 3-day-old seedlings. The number of PCR cycles (c) used is indicated to the right of the gel. (C) Germination of wild-type Col-0 seeds in the presence of 30 µM estrogen and different concentrations of PAC. No significant differences between genotypes were present.

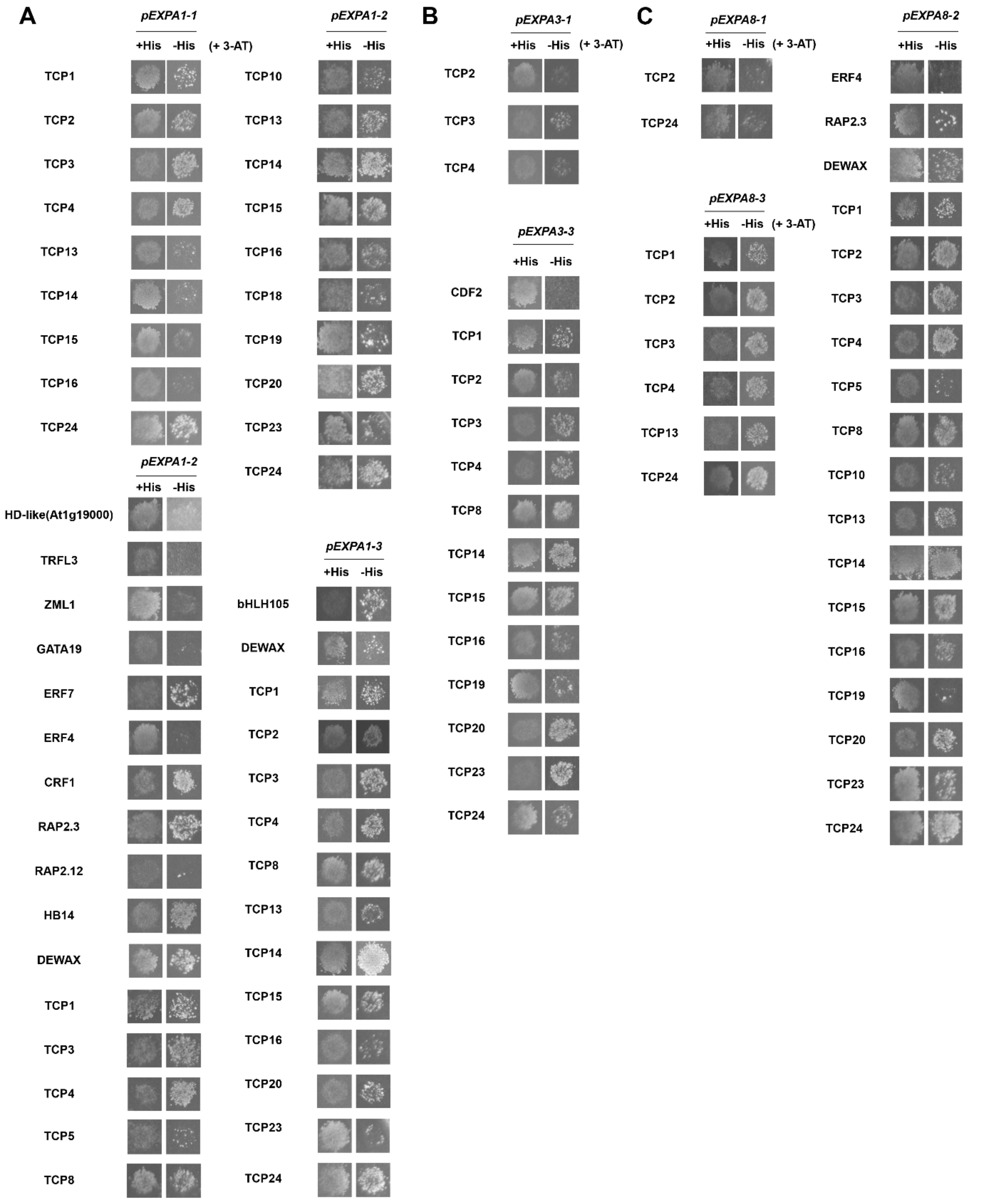

Figure S2. Y1h screening of *EXPA* promoter fragments. Related to Figure 2. (A) Colonies showing yeast 1-hybrid interactions between TFs and *EXPA1* promoter fragments. (B) Same as (A) showing for the *EXPA3* promoter, and (C) for the *EXPA8* promoter. The promoter fragment used as bait is indicated. Image panels on the left show yeast growth on SD-Leu-Trp media, and on the right SD-Leu-Trp-His (+ 3-AT as required).

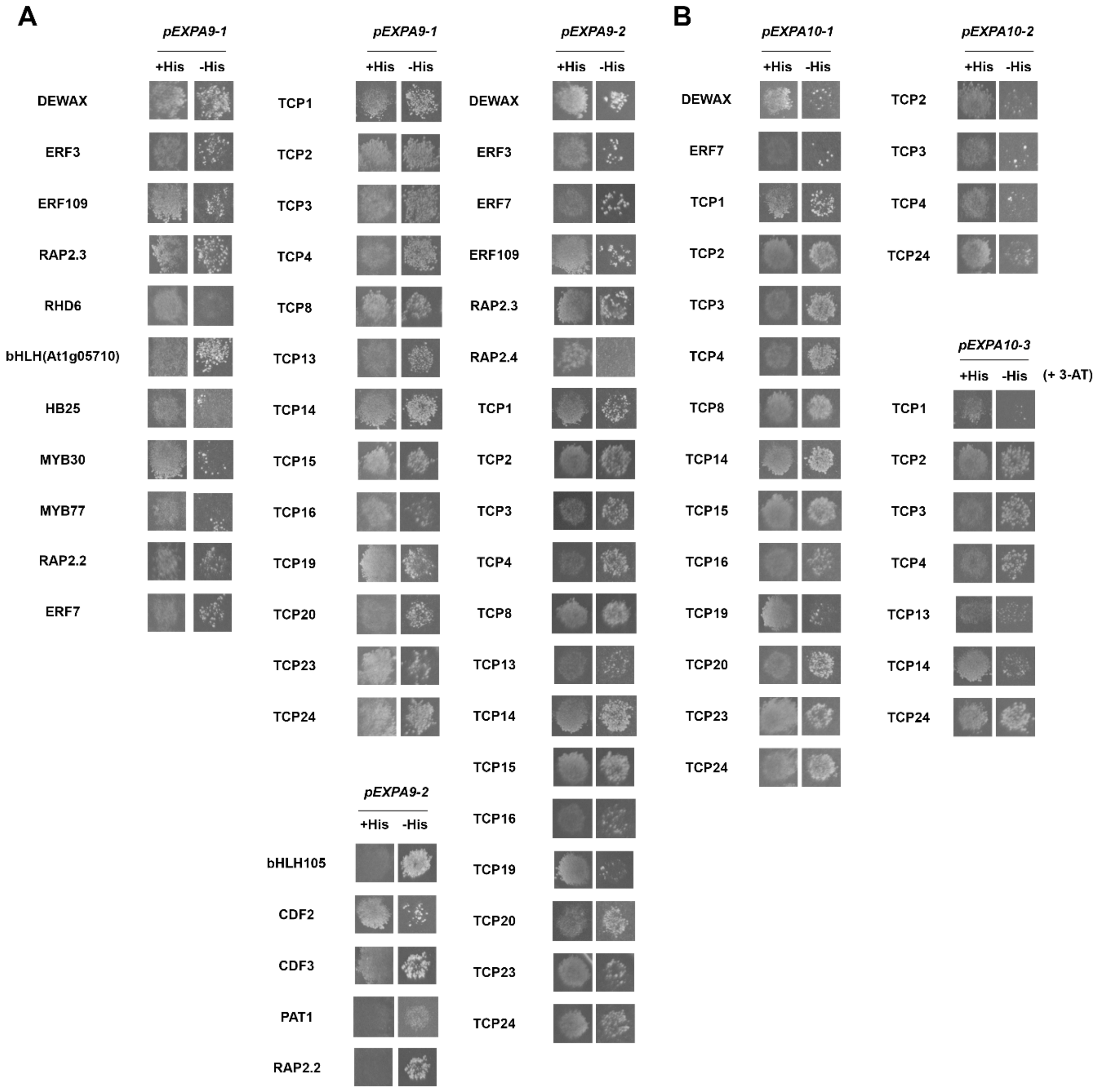

Figure S3. Y1h screening of *EXPA* promoter fragments. Related to Figure 2. (A) Colonies showing yeast 1-hybrid interactions between TFs and *EXPA9* promoter fragments. (B) Same as (A) showing for the *EXPA10* promoter. The promoter fragment used as bait is indicated. Image panels on the left show yeast growth on SD-Leu-Trp media, and on the right SD-Leu-Trp-His (+ 3-AT as required).

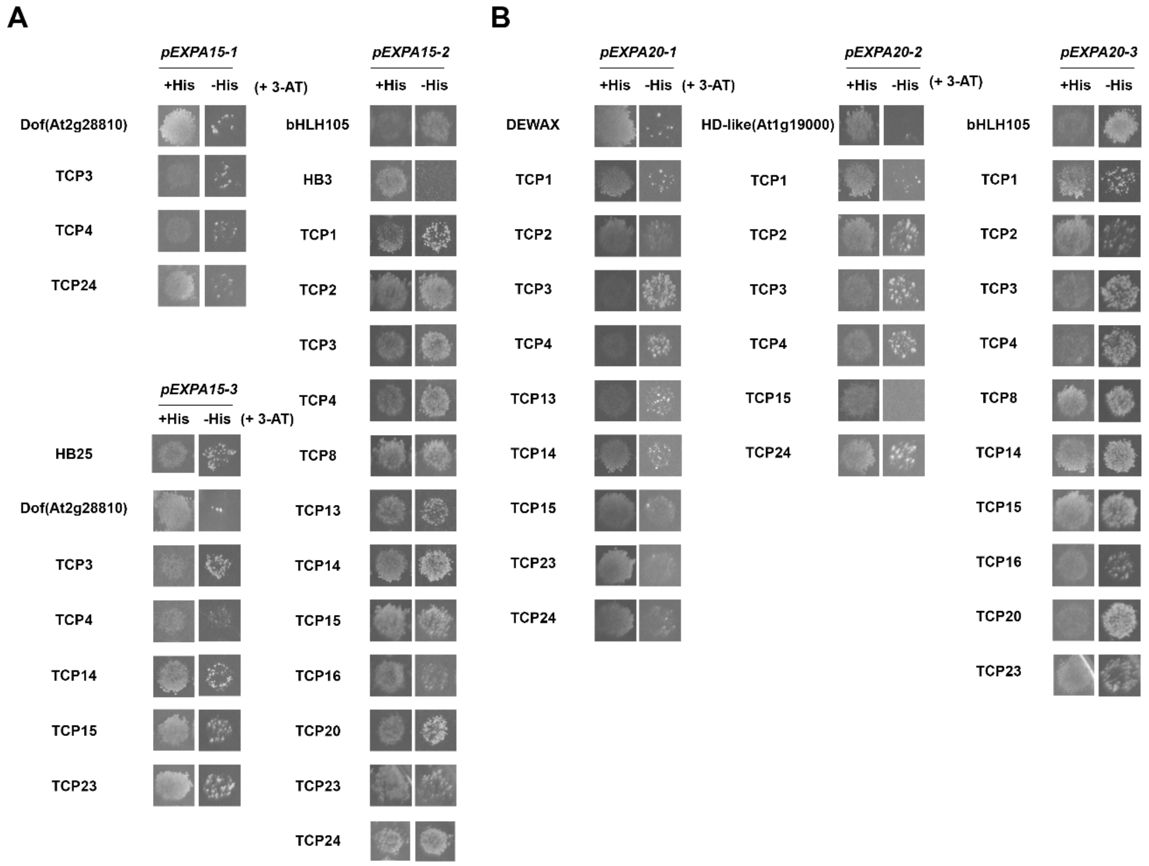

Figure S4. Y1h screening of *EXPA* promoter fragments. Related to Figure 2. (A) Colonies showing yeast 1-hybrid interactions between TFs and *EXPA15* promoter fragments. (B) Same as (A) showing for the *EXPA20* promoter. The promoter fragment used as bait is indicated. Image panels on the left show yeast growth on SD-Leu-Trp media, and on the right SD-Leu-Trp-His (+ 3-AT as required).

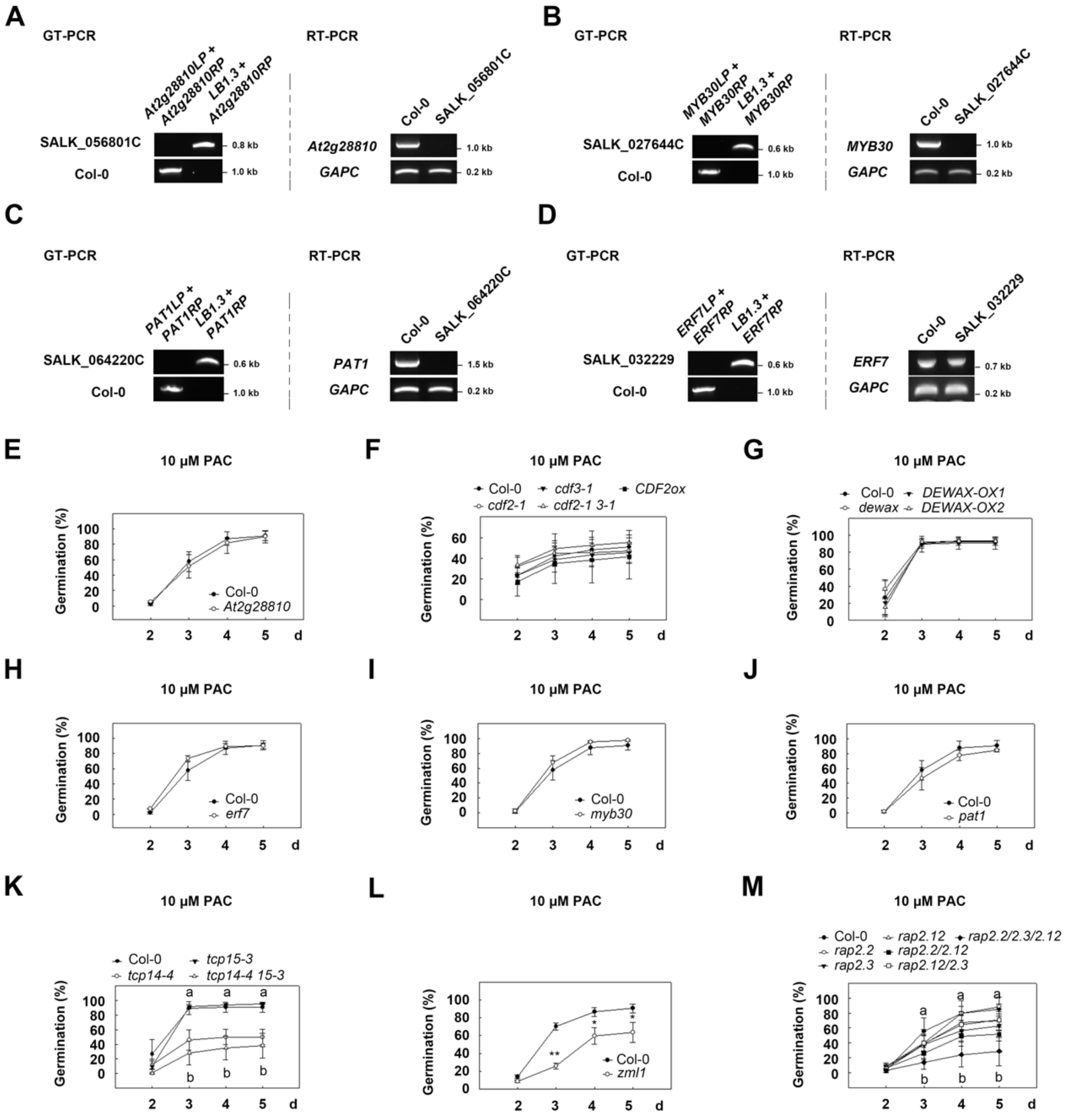

Figure S5. Phenotyping DELLA and EXPA-binding TF mutant seeds. Related to Figure 5. (A) Genotyping PCR using genomic DNA confirming the T-DNA insertion in *At2g28810* (GT-PCR), and the lack of transcript for this gene using RT-PCR. (B) Same as (A) for *MYB30*. (C) Same as (A) for *PAT1*. (D) Same as (A) for *ERF7*. (E) Germination of *At2g28810* mutant seeds on 10 μM PAC. (F) Same as (E) for *cdf2 cdf3* mutant seeds. (G) Same as (E) for *dewax* mutant and overexpressing seeds. (H) Same as (E) for *erf7* mutant seeds. (I) Same as (E) for *myb30* mutant seeds. (J) Same as (E) for *pat1* mutant seeds. (K) Same as (E) for *tcp14* and *tcp15* mutant seeds. Different lowercase letters indicate statistically significant differences (one-way ANOVA with Tukey post hoc test, $P < 0.05$). (L) Same as (E) for *zml1* mutant seeds. Asterisks indicate significant pairwise differences in comparison to the wild type control using Student's *t*-test (*, $P < 0.05$; **, $P < 0.01$). (M) Same as (E) for *rap2.2 rap2.3* and *rap2.12* mutant seeds. Different lowercase letters indicate statistically significant differences (one-way ANOVA with Tukey post hoc test, $P < 0.05$).

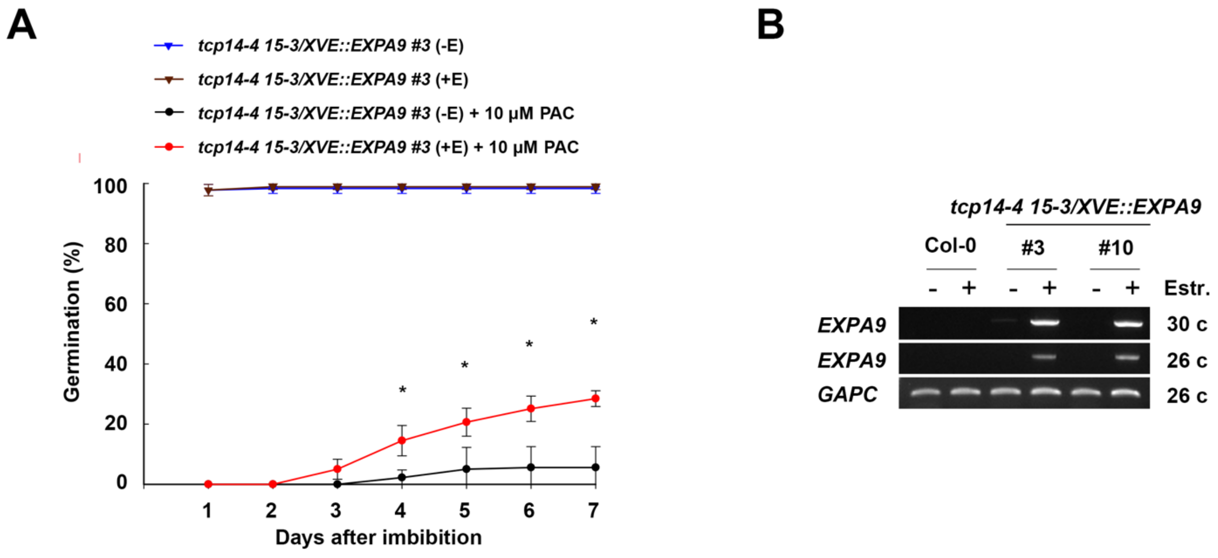

Figure S6. Complementation of the *tcp14 tcp15* phenotype by *EXPA* expression. Related to Figure 6. (A) Germination of *XVE::EXPA9* in the *tcp14-4 tcp15-3* line 3 on water and 10 μM PAC in the absence (-E) and presence (+E) of 30 μM estrogen. (B) RT-PCR confirming the induction of *EXPA9* in response to estrogen application in two independent lines of *tcp14-4 tcp15-3/XVE::EXPA9*. 3-d-old seedlings were used for RNA extraction.
